# WormAssay: A Novel Computer Application for Whole-Plate Motion-based Screening of Macroscopic Parasites

**DOI:** 10.1371/journal.pntd.0001494

**Published:** 2012-01-31

**Authors:** Chris Marcellino, Jiri Gut, K. C. Lim, Rahul Singh, James McKerrow, Judy Sakanari

**Affiliations:** 1 Sandler Center for Drug Discovery, University of California San Francisco, San Francisco, California, United States of America; 2 Department of Computer Science, San Francisco State University, San Francisco, California, United States of America; 3 Case Western Reserve University School of Medicine, Cleveland, Ohio, United States of America; McGill University, Canada

## Abstract

Lymphatic filariasis is caused by filarial nematode parasites, including *Brugia malayi*. Adult worms live in the lymphatic system and cause a strong immune reaction that leads to the obstruction of lymph vessels and swelling of the extremities. Chronic disease leads to the painful and disfiguring condition known as elephantiasis. Current drug therapy is effective against the microfilariae (larval stage) of the parasite, but no drugs are effective against the adult worms. One of the major stumbling blocks toward developing effective macrofilaricides to kill the adult worms is the lack of a high throughput screening method for candidate drugs. Current methods utilize systems that measure one well at a time and are time consuming and often expensive. We have developed a low-cost and simple visual imaging system to automate and quantify screening entire plates based on parasite movement. This system can be applied to the study of many macroparasites as well as other macroscopic organisms.

## Author Summary

The World Health Organization estimates that there are approximately 37 million people who are afflicted by *Onchocerca volvulus* (the parasitic worm that causes river blindness) and over 120 million people afflicted by the filarial worms *Wuchereria* and *Brugia* spp. (causative agents of lymphatic filariasis or elephantiasis). Current mass drug administration includes albendazole and either diethylcarbamazine or ivermectin. These drugs, however, are effective at killing the early larval stage (microfilariae) released from adult female worms but they do not kill the adult worms. Adult worms can live up to 10 or more years, releasing thousands of microfilariae per day. It is essential therefore to treat infected individuals with macrofilaricides in order to prevent the adult parasites from producing microfiliariae for the duration of the infection and to treat the disease. In order to screen candidate drugs for use as macrofilaricides, we have developed an inexpensive system and simple method for quantifying the effectiveness of drugs on parasite movement. The apparatus uses a commodity video camera, a computer and a newly developed free and open source software application to provide automated and quantitative measurements of parasite motility on each plate of worms. This system is not only useful for high throughput screening of macroparasites but can also be applied to the study of other macroscopic organisms as well.

## Introduction

Lymphatic filariasis is a devastating parasitic disease that affects more than 120 million people in 81 countries [1]. Also known as elephantiasis, the disease is caused by parasitic nematodes whose adult forms inhabit the lymphatic system. WHO estimates that over 1.3 billion people are at risk for lymphatic filariasis and approximately 95% of infected individuals live in Africa and South-East Asia.

Lymphatic filariasis is spread mainly by three species of nematodes in the family Filariodidea: *Wucheria bancrofi*, *Brugia malayi* and *Brugia timori*. The adult forms are threadlike roundworms from 2–5 cm in length and adult females produce millions of microfilariae that circulate in the blood where they are vectored by mosquitoes. The microfilariae develop into the infectious form in the mosquito and are inoculated into individuals when the mosquito takes a blood meal. Larval forms migrate to the lymphatic vessels and mature in 6–12 months and begin releasing microfilariae, completing the cycle of transmission.

The WHO currently advocates interrupting transmission of the disease via an annual mass drug administration of single doses of albendazole and either diethylcarbamazine or ivermectin. These drugs are effective at killing microfilariae but not effective against adult worms (macrofilariae). Since adult worms can live for up to 6–8 years, treatment must be given on a regular basis to break the cycle of transmission. This widespread treatment is logistically challenging and costly, particularly in endemic regions that are politically unstable. Such widespread application also raises the threat of resistance, whose first signs are being seen with ivermectin in *Onchocerca volvulus* [2]. Mass drug administration would be greatly aided by a macrofilaricidal drug, as the adult parasites would not be able to continue producing microfilariae for the duration of the infection. Hence, there is a great need for new macrofilaricidal drug candidates.

Currently there is no high throughput screening (HTS) method available to screen compounds targeting any of these macroscopic nematodes in vitro. Assays have been developed in recent years that score worm migration, feeding and development [3]–[19] as well as worm viability based on the MTT ((3-(4,5-dimethylthiazola-2-yl)-2, S-diphenyl tetrazolium bromide) assay [7], [8], [11]–[13], [20], [21], but these are not amenable to screening 1000's of compounds with a quick turnaround time on large worms such as filarid nematodes. Parasite movement is an important indication of the effectiveness of a drug and constitutes a crucial phenotype for HTS. Existing assays however, read only single wells at a time, are low-throughput and are unaffordable for many laboratories in developing nations [3], [22]–[24]. In the context of analyzing the phenotypes of model organisms, Buckingham and Sattelle published an algorithm for measuring the thrashing of *Caenorhabditis elegans* via a statistical analysis of the covariance matrix between sets of frames to determine the period of thrashing [24]. The algorithm is specific to the thrashing phenotype and is not a complete ready-to-screen software application. Both Buckingham and Sattelle's, and Ramot et al.'s [23] applications require that individual videos be (manually) recorded of each well and then be processed offline. Hence no affordance is made for automatically labeling the output data, either by well or using plates barcodes. This makes their tools, as currently implemented, unsuitable for use in a medium- or high-throughput assay. Smout et al. describes an apparatus that does not use an optical assay but instead uses special microtiter plates (xCELLigence and E-plate, Roche Inc.) to measure movement [17]. Recently, efforts have been made for screening *Schistosoma* spp. based on the quantification of multiple phenotypic responses of the parasites to drugs (as opposed to motility or a biochemical activity) [25]. However, these methods are also not yet available as a ready-to-screen software application or generalized to other macroparasites.

We have developed an inexpensive system (“WormAssay”) for quantifying parasite movement based on worm motility. The apparatus uses a commodity video camera, computer and a newly developed free and open source software application to provide quantitative measurements of parasite motility on entire plates. The application can process multiple wells simultaneously without user interaction, and automatically identifies each well in the plate and labels the output data accordingly. This system can be used to assay large parasites such as the filarid nematodes as well as other macroparasites. WormAssay's automation of the video capture step and lack of need for any interaction with the computer software during scoring differentiates it from all existing motion-based schemes, and permits current screening of 400 worms per assay each week.

## Materials and Methods

### *Brugia malayi* assays

Individual adult *Brugia malayi* female worms (TRS Labs Inc., Athens, GA) were assayed in RPMI-1640 (25 mM HEPES, 2 g/L 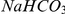, Antibiotic/Antimycotic, 5% HI FBS) in 24-well tissue culture plates (1 worm/well). 30 mM stock solutions of albendazole (methyl 5-(propylthio)-2-benzimidazolecarbamate, Sigma), ivermectin (22,23-dihydroavermectin B1, Sigma) and fenbendazole (methyl 5-(phenylthio)-2-benzimidazolecarbamate, Sigma) were prepared with DMSO (Sigma) and serially diluted in media into concentrations of 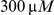, 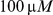, 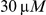, 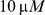, 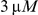. DMSO was used as the control and each concentration was run in triplicate. Plates were maintained in a 37

C 5% 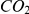 incubator for 48 hours. 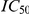 data were calculated using Microsoft Excel (Microsoft Corp.) and Prism 5 (GraphPad Software, Inc.).

### WormAssay computer program

The assays were performed using the open source computer software program described here. This program is named WormAssay. Plates were visualized using a Canon HV-40 Vixia HDV camcorder (Canon Inc.) providing 1080p H.262/MPEG-2 Part 2 compressed HDV video connected via IEEE1384 to an Apple iMac with a 2.93 GHz Intel Core i7 4-core CPU (Apple Inc.) and WormAssay (version 0.15) for 1 minute. The application and source code are available for free use, modification and redistribution under the terms of the GNU Public License (version 2 or later; see http://www.gnu.org/licenses/gpl-2.0.html). The application and its source code can be downloaded from http://code.google.com/p/wormassay/.

## Results

**Figure 1:**
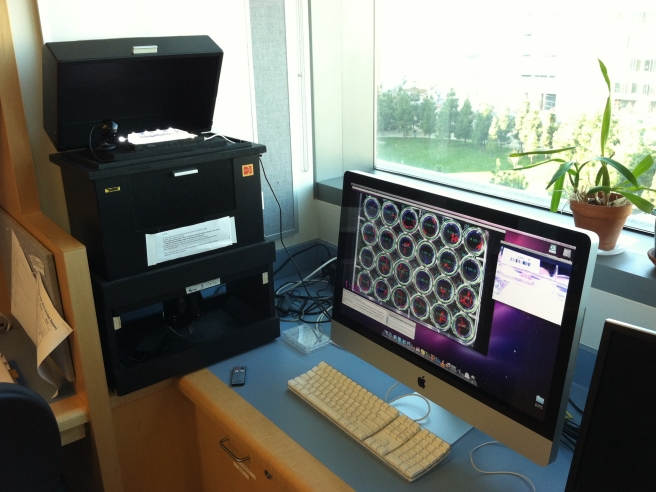
Dark field macroscopic imaging apparatus and computer application. The video camera is positioned below the microtiter plate and the plate is recorded using the WormAssay software.

**Figure 2:**
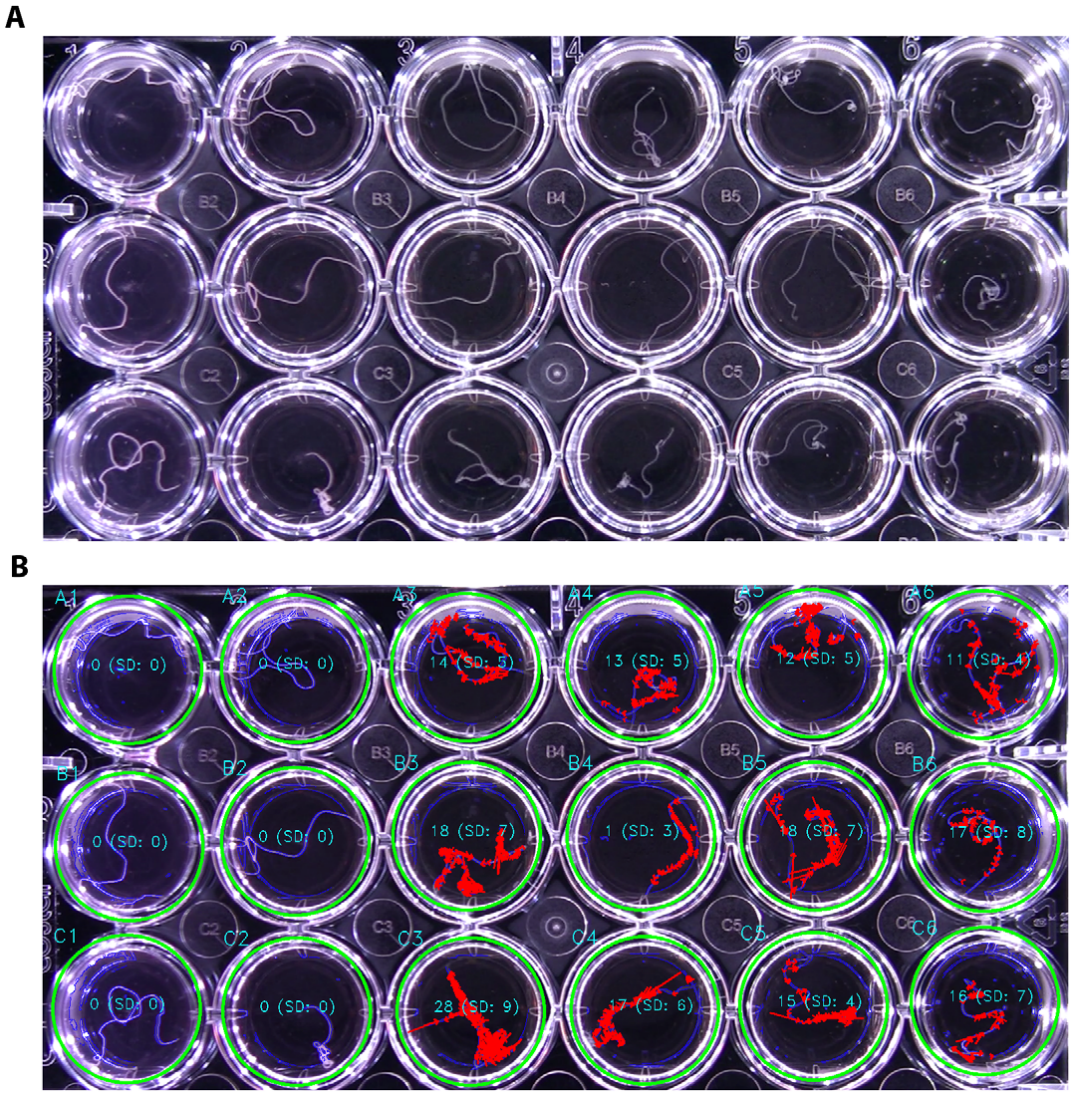
Microtiter plate images and application real-time preview. (A) Video frames of 24-well plates of *Brugia malayi* were recorded using the apparatus. (B) Screen capture of the software's real-time preview user interface. Green circles indicate that the wells have been detected and that the program is ready to begin recording data. The blue outline in each well is the worm (or other well artifacts) and the red color indicates the worm's movements. Mean movement units are measured in real-time and are shown for each well using the Lucas-Kanade Optical Flow algorithm. Canonical well labels determined by the well finding algorithm are drawn in teal in the preview image by the application. (There is no movement in the 2 left columns because the worms are dead due to high drug concentrations.)


**Figure 3:**
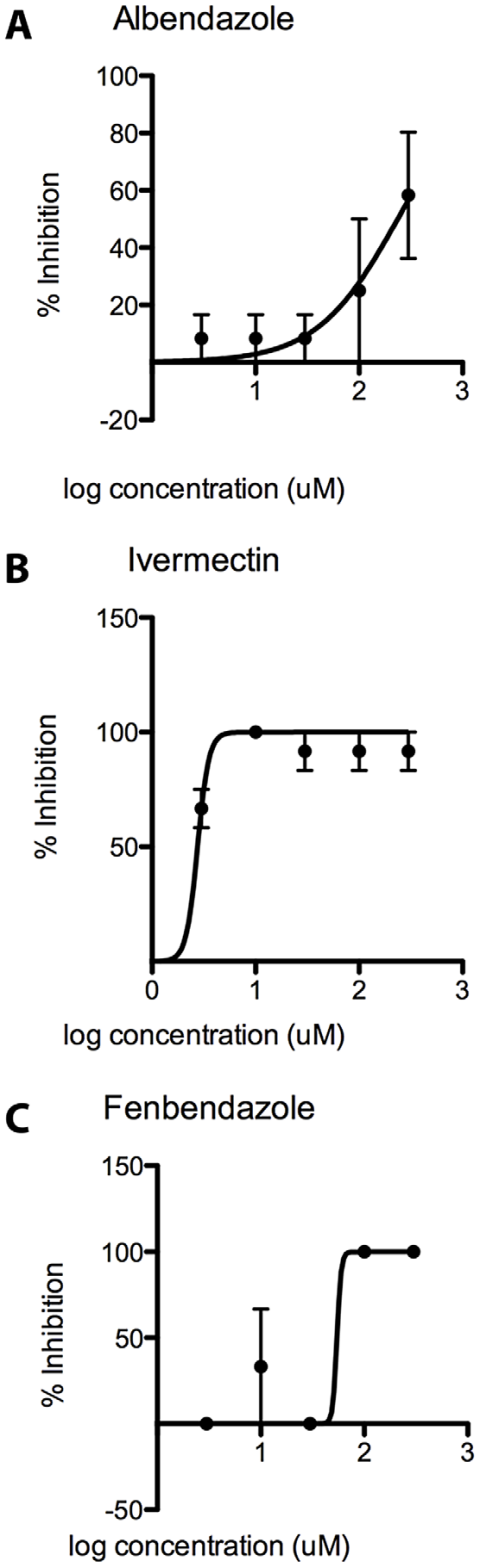
Dose-response curves of anthelmintic compounds used for validation. 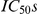
 of (A) albendazole (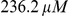), (B) ivermectin (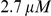) and (C) fenbendazole (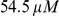) determined using the proposed method with compounds at concentrations of 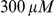, 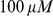, 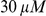, 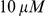, 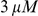 and 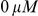.

The WormAssay was developed for use in high throughput screening of *Brugia malayi* adult female worms in 24-well plates. Plates were screened using the visual imaging system (Figure 1 and 2) and software application to assess drug effects on adult female *Brugia malayi*. One minute video recordings using the Lucas-Kande Optical Flow algorithm (see Analysis Algorithms) were taken of each plate and mean optical flow movement units for each worm were converted to percent inhibition: 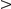
15 movement units = 0%, no inhibition (worms are very active); 10–15 movement units = 25%, slight inhibition (worms are active); 5–10 movement units = 50%, moderate inhibition (worms slightly moving); 2–5 movement units = 75%, good inhibition (worms barely moving); 0–2 movement units = 100%, very effective killing (worms are dead), using Microsoft Excel and the CSV files generated by WormAssay.

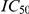
 data for albendazole, ivermectin and fenbendazole were calculated for each compound after 48 hours of incubation (see Figure 3 and Videos S1, S2 and S3). Visual analysis of video recordings and 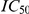 data indicate that ivermectin (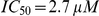) was the most effective in killing worms compared to albendazole (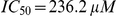) and fenbendazole (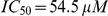). After 48 hours, only control worms (1% DMSO only) were highly active. In the ivermectin plate, only one of the 3 replicate worms assayed in 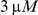 was found to be barely moving while the other 2 were not moving at all. Worms assayed with albendazole, however, were found to be active even at 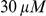. After 48 hours of incubation at 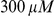, one of the 3 worms exhibited activity after 48 hours, while the second replicate was barely moving and the third replicate was dead. Indeed, the 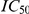 of albendazole was 100-fold higher compared to that of ivermectin. Worms assayed with fenbendazole at 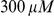 and 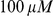 were all dead but worms in 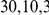 and 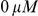 concentrations were found to be active after 48 hours.

### User interface

WormAssay's data acquisition does not require user interaction or configuration and is suitable for robotics integration with any multi-degree-of-freedom plate manipulator. Data acquisition automatically begins when a plate appears in the field of view of the camera and data is written immediately upon removal of the plate (Videos S4, S5 and S6). Video recordings of each read are archived. Motility and other assay data are written to CSV (spreadsheet style) files for use with standard statistical analysis software tools. Barcode reading is performed on the video stream (or from another video camera attached) to automatically label results. The application can automatically email results at the end of a run, for example, when used in a unattended automated assay.

### Plate tracking state machine

**Figure 4:**
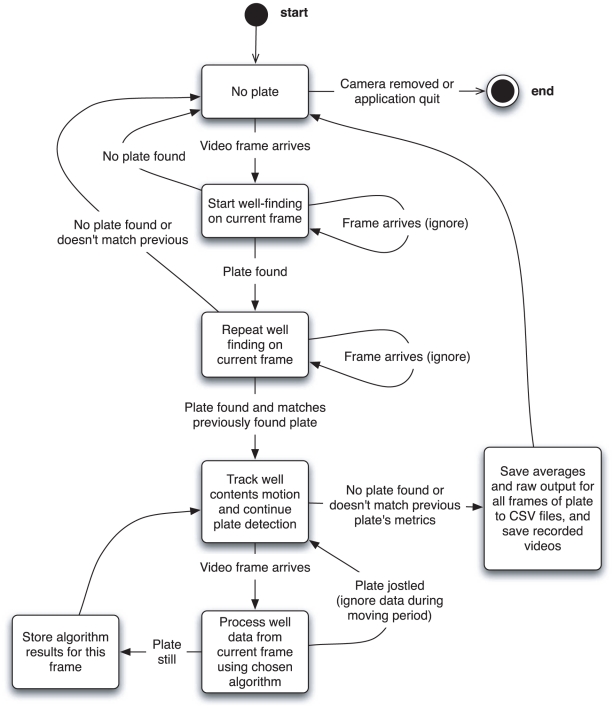
Simplified state machine of the plate detection and tracking logic. The nodes indicate distinct states (or the start/end pseudostates) and the edges indicate transition conditions.

An extended finite state machine [26] is modeled programmatically for each connected camera (see Figure 4). The finite state machine describes the continuous analysis logic of WormAssay that is used to automatically start recording data when a plate is presented to the camera's field of view. If more than one camera is attached to the computer, only the first plate identified on a camera is used for analysis. Other cameras are ignored until that read is complete and the plate is removed from the field of view, except for the purpose of plate identification, where all cameras are inspected simultaneously for common barcode formats. This allows one camera to be used for the motility assay and one or more other cameras to be used for optional barcode recognition. Any barcode text found is used to label the plate output in the CSV files that are output containing the motility data.

### Well finding

The application has no knowledge of the specific geometry of the microtiter plates, except for the number of wells in each row and column of the supported plate sizes. This allows for great tolerance in terms of specific plate geometry and in the position of the plate within the camera's field of view. This is contrary to the scheme used by most microtiter plate assay equipment, where a mechanical sensor is positioned over or in the well of interest, one well at a time.

**Figure 5:**
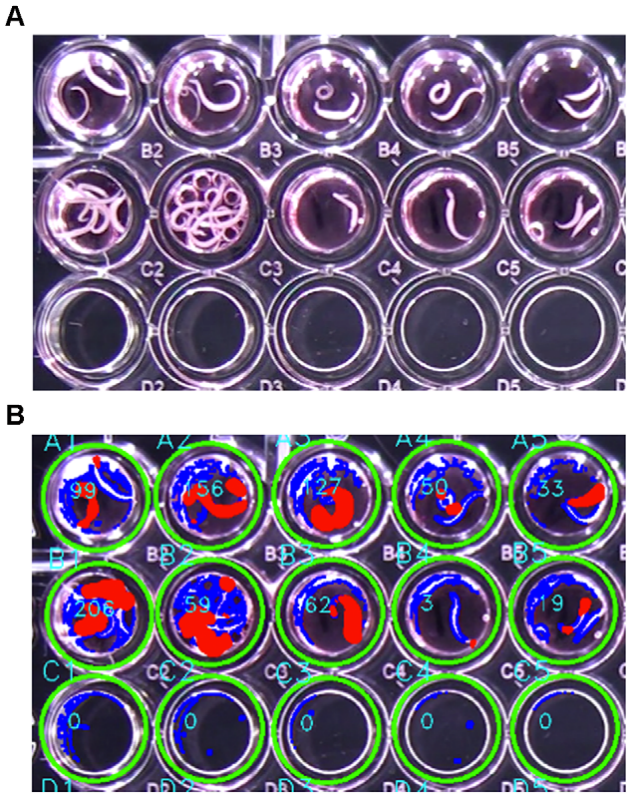
*Schistosoma mansoni* adult worms can also be assayed in 24- or 96-well plates using the WormAssay visual imaging system. (A) Unprocessed video frame in a 96-well plate. (B) Screen capture of the software's user interface for those plates using the Consensus Voting Luminance Difference algorithm (see caption for Figure 2b).

The WormAssay well finding algorithm iterates through acceptable plate configurations in parallel, corresponding to 6-, 12-, 24-, 48- and 96-well microtiter plates. First, Canny's algorithm [27] is used to find edge features. The Hough (circle) transform [28] is used to find candidate wells efficiently among these edge features. The search for appropriate circles is performed efficiently by limiting the candidate circle size to correspond to the expected range of sizes for a well, under the assumption that the plate fills a simple majority of the imaging field. The resulting circles are then filtered to only accept those that lie on an axis-aligned collinear grid corresponding to the plate row and column configuration. If all expected wells are then found for the plate configuration, the algorithm deems the plate found and moves the state machine down the found edge. If a plate is not found, then the frame image is preprocessed by linear amplifying the high-frequency components of the image. This makes it possible for the Canny edge finding step of the Hough transform to detect the edges that correspond to the well circles in out of focus or poorly illuminated images. If a plate has already been detected, future detection attempts only search for that plate configuration (well count), to reduce CPU resource utilization. Finally, the application uses the location of the wells to label each well canonically (e.g. A1, B1, etc.) in both the real-time screen preview (teal colored text in Figure 2b and 5b, also see Videos S4, S5 and S6) or in the output CSV file data.

### Analysis algorithms

We developed two analysis algorithms. The first determines the average velocity of the moving contours inside each well. This algorithm derives the velocity from the optical flow vectors of the luminance component of the video stream from a pair of adjacent frames approximately 100 ms apart. The algorithm uses the sparse iterative version of the Lucas-Kanade optical flow in pyramids provided with the OpenCV framework [29]–[31]. The set of pixels to be considered when calculating the optical flow rate in the current frame is limited in order to make the analysis computationally feasible. This set of points is chosen at random from the set of points that lie on the edge contours found within the well using Canny's algorithm (colored blue in Figure 2b and 5b). Some of these points will correspond to the well itself or other artifacts, but these pixels will not possess a positive optical flow, and will have no impact on the result, as the rate is only determined based on the pixels which are determined to be moving.

This algorithm is useful for scoring rates of motion (or lack thereof) of single parasites with high accuracy as it can reliably differentiate small differences in velocity which may correspond to differing amounts of motility inhibition. A velocity in single dimensional pixel units per second is reported. Only moving components are considered, so this assay is not suitable for assays where a combination of dead (motionless) and moving parasites are present in a single well, since only the moving parasites will be considered in the score. This algorithm is described in WormAssay's Options-Analyzer menu as “Lucas-Kanade Optical Flow.”

The second algorithm is an algorithm that detects changes in the occupation and vacancy of pixels between a group of frames. It uses difference information between a subset of 5 frames chosen at random from the frames that arrived in the past second. First, a difference is performed on each of the 3 color channels of each of the 5 frames and the current frame. Then high frequency components are removed from each of the set of 5 difference values. A voting scheme is employed to determine when a pixel has had its contents changed. Three or more changed pixels is deemed a quorum, otherwise the changes are ignored and deemed noise. The number of filled or vacated pixels is then summed and taken as a fraction of the total number of pixels within the well's circle (times 1000 to improve numerical readability). This number is reported as an arbitrary area unit indicating motility. This algorithm is useful for detecting very low levels of movement or for quantifying the aggregate movement of more than one parasite in a given well. This algorithm is described in the application as “Consensus Voting Luminance Difference.”

All algorithms process in real-time, in parallel on each well. The algorithm programming model is extensible; new algorithms can be added independently of other components of the application. To avoid recording spurious values when the plates are being moved at the beginning or end of a run, the software ignores any frames whose total motion (via the pixelwise mean of the simple interframe absolute difference across all color channels) exceeds a threshold. This value may need to be modified for assays with very large or motile organisms. This is the only non-general threshold used in the application. Improving this aberrant (whole plate) motion detection is a possible area of further research.

Both algorithms (and the well detection) are computationally intensive, and are not able to process every frame of the 1080p (1080×1920 pixels) video input, which is typically 24 or 30 frames per second. On a modern (2011) typical multicore desktop computer, we are able to process 5–10 frames per second, which yields satisfactory results. Since recording of all wells is done in parallel, this is significantly faster than the 5–10 minute recoding times necessary to generate even short 10 frames per second movies on a well-by-well basis on commercial plate microscopes (e.g. on the GE IN Cell Analyzer 2000.)

### Dark-field macroscopic imaging apparatus

We also developed a dark-field parallel macroscopic imaging apparatus connected to an HDV camera with an IEEE1394 interface using inexpensive materials [32] (Figure 1). The apparent observable quality of the video recordings improves with greater levels of contrast of the worm with the background. A dark-field imaging scheme provides the most striking contrast, which gives the computer application a greater level of signal-to-noise to analyze. We found that a dark-field imaging scheme, where the plates are illuminated from the side with a uniform light source (in our case white LEDs) and a dark backdrop at least 5 cm from the focus plane of the plate, provides ideal images for recording and analysis. Due to the well finding mechanism, the plates must fill the majority of one of the imaging axes, although there is considerable room for error.

The apparatus used consists of a light-tight box with a hinged lid on the top, with the video camera mounted outside (to ensure easy access and proper cooling) at the bottom of the box and recording upwards. The whole box is made of plywood with some metal parts, all painted black to minimize reflections. The plate is positioned above the camera at such a distance that allows the plate image to fully fill the field of view (approx. 35 cm). The plate is illuminated by a dimmable white LED strip (Home Accent Lighting Kit, White, PPA International) mounted parallel with the plate walls at a distance of 25 mm.

The assay is very sensitive to inadvertent plate motion and illumination that moves or is poor. Hence, it is important to shield the recording field from ambient light so that the operator's movement does not cast a moving shadow on the field of view.

## Discussion

One of the major stumbling blocks in identifying candidate drugs for the treatment of lymphatic filariasis and river blindness is the lack of a high throughput screening system for these large worms. The filarid nematodes are long and threadlike and cannot be easily assayed in a 96-well format. We therefore developed an automated imaging system in which *Brugia malayi* could be assayed in 24-well plates using a simple and inexpensive method called the WormAssay.

The WormAssay is a visual imaging system that utilizes a novel software program to capture video recordings to assay the effect of compounds on macroparasites. To test the robustness of the software program, we assayed *Brugia malayi* female worms with 3 antihelminthic drugs: albendazole and fenbendazole (benzimidazoles) and ivermectin (macrocyclic lactone). Albendazole is widely used to treat intestinal nematode infections including ascariasisis and hookworm; ivermectin is used in mass drug administration to treat filariasis; and fenbendazole is used in the veterinary field to treat animals infected with intestinal parasites. 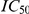 data using these compounds with adult filarids are not available. However, the study by Tompkins, Stitt and Ardelli (2010) showed that when adult male and female *Brugia malayi* were exposed to ivermectin at 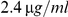 for 3 days, the motility (as measured in movements/minute) decreased from 250 units to 125 units which is approximately equivalent to the 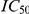 (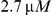) in our study using ivermectin (molecular weight = 875.1 u) for 2 days [9]. Townson et al. (1990) showed that adult male *Onchocerca gutturosa* exposed to 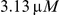 of ivermectin in the course of 7 days had greatly reduced motility levels (based on mean motility scores from 0–10) compared to controls [13]. Motility scores for male worms exposed to 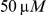 albendazole for 2 days were similar to those for their control worms. Although Townson et al. used *Onchocerca* adults in their study, their results for both albendazole and ivermectin are consistent with our data.

We are currently using the visual imaging system to screen approximately 400 adult *Brugia* females per assay. Along with our visual imaging system, we use a Biomek FX (Beckman Coulter) instrument to remove media from each well and dispense compounds. It takes approximately 15 minutes per plate of 24 worms (1 worm/well) to screen compounds at a single concentration and approximately 20 minutes per plate for an 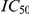. The system is capable of screening more worms but assay throughput is currently limited by the number of worms produced and delivered. Once we receive the 24-well plates containing individual adult female *Brugia*, we estimate that it takes one person approximately 6–7 hours to setup an assay to screen 96 compounds (at single concentrations) using the Biomek FX and run the WormAssay (on Day 0). Plates are assessed every day for 3 days using the visual imaging system which takes approximately 15–20 minutes for 16 plates. Control worms under these conditions remain highly active while worms treated with low micromolar concentrations of ivermectin are killed as evidenced by the lack of motility. We have observed that the lack of motility is correlated with worm death; dead worms appear more opaque (in some cases are slightly tanned) and never regain motility.

Rather than using laborious and subjective methods of analyzing plates (manual examination of individual wells and plates with a dissecting scope and scoring worm movements relative to control worms), the WormAssay quantified each worm's movement simultaneously on the entire plate, with each plate taking approximately 30 seconds to 1 minute to read. Given the short read times, researchers can increase the number of replicates per compound, thus increasing the accuracy of the assay. Currently, the system requires an individual to place the plate into the visual imaging box but this system is amenable for use with a robotic arm, removing and replacing plates to and from a plate hotel. The software application also includes bar code reading capabilities and can easily be exported to spreadsheets for data analysis.

WormAssay is a unique high-throughput screening motility assay that performs a parallel analysis on each well of entire plates simultaneously, but is independent of specific plate geometry and parasite morphology. The application supports 6-, 12-, 24-, 48- and 96-well plates. WormAssay does not track specific organismal characteristics so it can assay the motility of a large range of macroscopic organisms that can be cultured in a microtiter plate, but is capable of tracking very small or refined movements. The assay requires commodity computer equipment and is compatible with a variety of HD 1080p (or greater resolution) cameras and video capture interfaces. This low-cost and simple-to-use system can also be applied to other target organisms as well. Movements of other macroparasites, including adult schistosome worms were also assessed (see Figure 5), and studies with other macroorganisms are currently being explored.

In summary, the WormAssay offers several advantages: 1) it is inexpensive with costs of the video camera, LED lights and camera totaling less than $3,000 USD and the software is freely available, 2) it is easy to use, i.e. the plate can be quickly placed into the box housing the video camera and removed, 3) video recordings are saved onto the computer along with the data and can be reanalyzed at a later time, 4) entire plates with 6-, 12-, 24-, 48- and 96-wells can be assayed simultaneously, 5) the phenotype (worm movement) is quantified and stored as CSV files and 6) can be more generally applied to the study of macroparasites or other macroscopic organisms.

## Supporting Information

Video S1 **Video recording generated from anthelmintic assay for albendazole.** The videos in supplements 1–3 may be re-scored by using the application's File–Open For Testing menu.
(MP4)

Video S2 **Video recording generated from anthelmintic assay for ivermectin.**
(MP4)

Video S3 **Video recording generated from anthelmintic assay for fenbendazole.**
(MP4)

Video S4 **User interface recording generated from anthelmintic assay for albendazole.** Supplements 4–6 are examples of the real-time user interface feedback provided by the application using the videos from supplements 1–3. The high frame drop rate observed is an artifact of the screen video recording (for the purposes of generating this supplement) and did not occur during the original data acquisition.
(MP4)

Video S5 **User interface recording generated from anthelmintic assay for ivermectin.**
(MP4)

Video S6 **User interface recording generated from anthelmintic assay for fenbendazole.**
(MP4)
